# Iodine—An Antidote to the Poison of the Rattlesnake

**Published:** 1854-04

**Authors:** E. Harwood

**Affiliations:** Joliet


*Iodine—an Antidote to the poison of the Rattlesnake.*

Messrs Eds:—I see from the February No. of your Journal and also from the newspapers, that Prof. Brainard of Rush Medical College (now in Paris) claims to be the first discoverer, that Iodine is an antidote to the poison of the Rattlesnake. The fact may be, and probably is original with him. But I deny that he was the first to discover it, unless he made the discovery previous to the 14th day of September, 1848, for it was at this time that I made the first application of Iodine to the human subject, for the purpose of neutralizing the poison of the Rattlesnake, and with entire success, on a boy about 15 years of age, residing in this neighborhood. I saw him him about six hours after he was bitten and found the limb (leg) somewhat œdematus; made external application of Tinct. Iodine, with camel hair pencil, and gave 20 drops internally once in four hours until swelling subsided. After a few doses and applications the boy recovered without any other treatment.

I have since used it soon after the wound was made, externally and alone, with the same result, and I am now perfectly satisfied that the Iodine treatment, thoroughly carried out, will deprive the Snake of all his terrors.

I do not deem it necessary to inject the Iodine into the tissues in order to obtain its beneficial results. Because the alcoholic tincture which I use will be as readily and more perfectly absorbed than the poison itself. And I know the tinct. to be much better than the Iodid. of Potass. or Aqueous solution, for the reason that it is more readily absorbed by applying to the surface of the body than any other preparation of the Iodine.

Yours, &c.

JOLIET, April 10, 1854. E. HARWOOD, M. D.

[In Vol. 1st of the North-Western Medical and Surgical Journal. page 396, is an article by Dr. Whitmire of Metamora, Illinois, in which he reports a case in his practice in June, 1846, treated by the external application of Tinct. Iodine. If there be any honor in priority of use, it is due to Dr. Whitmire.

EDS. JOURNAL.]

